# Epidemiology and factors associated with amoebic liver abscess in northern Sri Lanka

**DOI:** 10.1186/s12889-018-5036-2

**Published:** 2018-01-10

**Authors:** Selvam Kannathasan, Arumugam Murugananthan, Thirunavukarasu Kumanan, Nilanthi Renuka de Silva, Nadarajah Rajeshkannan, Rashidul Haque, Devika Iddawela

**Affiliations:** 1 Department of Pathology, Faculty of Medicine, University of Jaffna, Jaffna, Sri Lanka; 2 Department of Medicine, Faculty of Medicine, University of Jaffna, Jaffna, Sri Lanka; 3 Department of Parasitology, Faculty of Medicine, University of Kelaniya, Ragama, Sri Lanka; 4 St Johns’ Park Medical Center, Sydney, Australia; 5 International Centre for Diarrhoeal Disease Research, Dhaka, Bangladesh; 6 Department of Parasitology, Faculty of Medicine, University of Peradeniya, Peradeniya, Sri Lanka

**Keywords:** Amoebic liver abscess, Amoebiasis, *Entamoeba histolytica*, Sri Lanka

## Abstract

**Background:**

Clinically diagnosed amoebic liver abscess (ALA) caused by *Entamoeba histolytica* has been an important public health problem in Jaffna district, northern Sri Lanka for last three decades. In order to draw up a control strategy for elimination of this condition, knowledge of its epidemiology and factors associated with this condition in the local context is vital.

**Methods:**

All clinically diagnosed ALA patients admitted to the Teaching Hospital, Jaffna during the study period were included in the study and the data were collected using an interviewer administered questionnaire. One hundred blood samples from randomly selected toddy (a local alcoholic drink consisting of the fermented sap of the Palmyrah palm) consumers and 200 toddy samples were collected. Toddy samples were cultured in Robinson’s medium to establish the presence of *Entamoeba histolytica* in the sample. Climatic data and the total toddy sales in the district were obtained from the Meteorological and Excise Departments respectively. A sub group of randomly selected 100 patients were compared with 100 toddy consumers who were negative for *E. histolytica* antibody to explore the potential risk factors.

**Results:**

Between July 2012 and July 2015, 346 of 367 ALA patients were enrolled in this study. Almost all patients (98.6%) were males with a history of heavy consumption of alcohol (100%). Almost all (94.2%) were within the age group 31–50 years. None of the cultured toddy samples grew *E. histolytica*. The monthly incidence of disease peaked in the dry season, matching the total toddy sales in the district. Age, type of alcohol and frequency of drinking were identified as potential risk factors whereas frequency of alcohol consumption and type of alcohol (consuming toddy and arrack) were identified as the independent risk factors. Moreover, the knowledge, attitude and practices towards ALA were poor among participants and the control group.

**Conclusions:**

Though the number of cases has declined in recent years, ALA still remains as an important public health problem in Jaffna district. The transmission route of *E. histolytica* leading to ALA has to be further explored. Moreover, greater awareness among the public who are at risk would be beneficial in order to eliminate the disease.

**Electronic supplementary material:**

The online version of this article (10.1186/s12889-018-5036-2) contains supplementary material, which is available to authorized users.

## Background

Amoebiasis, a parasitic condition, caused by *Entamoeba histolytica*, affects at least 50 million people globally and killing 100,000 individuals every year [1]. The most common extra intestinal manifestation of invasive amoebiasis is the Amoebic Liver Abscess (ALA), apart from its classical amoebic colitis. In the recent past, clinically diagnosed ALA has been one of the two most important parasitological public health problems (second only to malaria) in Jaffna, northern Sri Lanka [2–6]. Recently, we have confirmed that the cases of ALA were amoebic in origin [6]. Interestingly, it was observed by us [4, 5] and few others from Sri Lanka and elsewhere [3, 7–9] that most of the ALA patients had the history of consuming local alcoholic beverage, toddy (a local alcoholic drink consisting of the fermented sap of the palmyrah palm). Moreover, Hai et al. were able to demonstrate *Entamoeba histolytica* in 12.5% of cultured toddy in vitro [9].

In addition to the consumption of alcohol, poor living conditions, overcrowding, unhygienic practices [10], age, sex [11] and men who have sex with men [12] were identified as the major risk factors for ALA.

Although malaria has been under control in Jaffna district since 2002, cases of clinically diagnosed ALA have been continuously reported to the Teaching Hospital (TH), Jaffna. In order to draw up a control strategy for elimination of the condition, knowledge about disease burden, its local epidemiology, associated risk factors, seasonality of the condition in the local context and the knowledge, attitude and practice (KAP) towards the disease among those who are at risk, is essential.

Therefore, this study was carried out to examine the above factors in order to propose a control plan which is expected to prevent or minimize the transmission of *Entamoeba histolytica* in Jaffna district, northern Sri Lanka.

## Methods

### Study design and the study period

A longitudinal study was conducted among the clinically diagnosed ALA patients admitted to Jaffna TH, during the period from July 2012 to July 2015. Clinicians arrived at a clinical diagnosis mainly based on the patient’s history, clinical features and other investigations such as haematological parameters and mainly with the aid of ultrasonography. Out of 367 patients admitted during the study period, 346 consented for study (response rate - 94%). Further, to explore the associated factors, a sub study was conducted by comparing 100 randomly-selected, male patients with sex-matched controls of toddy consumers who were negative for *E. histolytica* antibody.

### Collection of epidemiological information and the knowledge, attitude and practice towards ALA data

Relevant data were collected from the patients and the control group using a pretested interviewer administered questionnaire. The questionnaire contained both open and close ended questions in the native language (Tamil). The questionnaire included the following information, a) general socio demographic data b) personal hygienic practices and alcohol consumption history and c) the knowledge, attitude and practices in relation to ALA.

### Collection of toddy samples

Five taverns were selected based on where most of the patients consumed toddy. Twenty toddy samples (250 ml each) from each tavern were collected on different days of the month. Further, 100 samples of fresh toddy (250 ml each), were also obtained just after collection from the palmyrah tree, at each particular location. All samples were brought to the laboratory and filtered through a sieve, centrifuged at the rate of 2500 rpm for 10 min and the supernatant was discarded. The pellet was used for further investigation.

### Monthly toddy sales in Jaffna district during the study period

Records of monthly toddy sales (in bottles) in the entire district throughout the study period was obtained from the Excise Department, Jaffna.

### Collection of meteorological data

Records of monthly rainfall, temperature and the relative humidity of Jaffna district for the study period (2012–2015) were also obtained from the Meteorological Department, Thirunelvely, Jaffna.

### Examination of toddy samples for the presence of amoebic cysts

Pellets obtained from centrifuged toddy samples were used for microscopic examination and cultured in Robinson’s medium [13]. Briefly, pelleted toddy samples were inoculated in the Robinson’s medium and incubated at 37 °C for 48 to 72 h and checked for motile trophozoites.

### Data analysis

Data were analyzed using SPSS statistical software version 16 and Win Pepi software version 11.62. In addition to describing the characteristics and hygienic practices of the patients admitted to TH Jaffna, randomly selected one hundred male patients were compared in the sub study with 100 male participants who were negative for *E. histolytica* serum antibody to explore the risk factors. Pearson’s, Chi –squared with Yates correction statistical tests were used and odds ratios were calculated to identify the potential risk factors.

## Results

### Amoebic liver abscess burden in Jaffna District

**Fig. 1:**
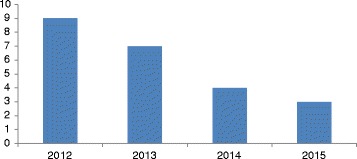
Number of clinically diagnosed ALA patients per 10,000 admissions at Teaching Hospital, Jaffna

In 2012, there were 9 ALA cases per 10,000 total hospital admissions which gradually declined to 3 cases per 10,000 hospital admissions in 2015 (Fig. 1).

### Socio-demographic characteristics of the patients

The condition showed a marked male predominance (98.4%). The large majority of patients (94.2%) were men of the age group 31–50 years. Most of them (77.6%) had studied up to G.C.E. Ordinary Level (secondary level of education) and the majority (64.5%) was labourers. Most of them (86%) stated that they earned a monthly income of LKR 5000–10,000 (USD 32.74–65.49) (Table 1).

**Table 1:** Socio demographic characteristics of ALA patients admitted to the Teaching Hospital, Jaffna compared with 100 individuals who were *E. histolytica* serum antibody negative

Variable	Categories	Patients (Cases)		*E. histolytica* serum antibody negative Individuals (controls)	
		*N* = 346	% with CI	*N* = 100	% with CI
Sex					
	Male	341	98.6(96.8–99.5)	100	100
	Female	05	1.4(0.5–3.2)	0	0
Age					
	20–30	0	0	18	18(11.4–26.4)
	31–40	110	31.8(27.0–36.1)	29	29(20.7–38.5)
	41–50	216	62.4(57.2–67.4)	23	23(15.5–32.0)
	51–60	20	5.8(3.7–8.6)	30	30(21.6–39.5)
Educational level					
	No school education	8	2.3(1.1–4.3)	2	2(0.03–6.5)
	Up to Grade 8	79	22.8(18.6–27.5)	24	24(16.4–33.1)
	Grade 6 to G.C.E O/L	258	77.6(69.8–78.9)	74	74(64.7–81.9)
	Followed G.C.E O/L	1	0.3(0.000–1.4)	0	0
Occupation					
	No Job	72	20.8(16.8–25.3)	5	5(1.9–10.7)
	Labourer	223	64.5(59.3–69.3)	66	66(56.3–74.8)
	Farmer	33	9.5(6.8–13.0)	19	19(12.2–27.6)
	Fishermen	17	4.9(3.0–7.6)	10	10(5.2–17.1)
	Self employed	1	0.3(0.000–1.4)	0	0
Monthly income					
	< LKR5000 (USD 32.76)	19	5.5(3.4–8.3)	3	3(0.8–7.9)
	LKR5000–10000 (USD 32.76–65.49)	298	86.1(82.2–89.5)	87	87(79.3–92.6)
	LKR10001-LKR 25000 (USD 65.49–163.72)	29	8.4(5.8–11.7)	10	10(5.2–17.1)

### Geographical distribution of the patients

Patients were more or less equally distributed within the 15 Medical Officers of Health divisions (basic health administrative divisions) in Jaffna district and the majority (75%) of them was from the rural area.

### Personal hygienic practices among the patients

The majority (93%) stated that they lived in households with water sealed toilets and most of them (88.4%) revealed that the distance between the toilet and the drinking water source was roughly about 50 feet (Table 2). However, it was observed there were no latrine facilities in most of the taverns. Further, all reported that they consumed non-purified water and the majority (77.5%) stated that they occasionally ate food that was not home-cooked. Moreover, most (98.3%) pointed out that they washed their hands with soap and water after defecation, but, 42.5% accepted that they rarely washed their hands with soap and water before meals (Table 2). Almost all of them (99.7%) stated that they cut their nails only rarely or at long intervals (Table 2).

**Table 2:** Personal hygienic practice toward ALA among patients compared with *E. histolytica* antibody negative (disease free) individuals

Variable	Categories	Patients (Cases)		*E. histolytica* serum antibody negative Individuals (controls)		Statistics
		*N* = 346	% with CI	*N* = 100	% with CI	
Latrine facilities	No facility/open air defecation	3	0.9 (0.2–2.3)	0	0	Pearson’s chi-square with Yates’s correction = 0.927 *P* = 0.336
	Using pits	21	6.1 (3.9–9.0)	3	3 (0.8–7.9)	
	Water seal latrine	322	93.1 (90.0–95.4)	97	97 (92.1–99.2)	
Distance between latrine and drinking water source	roughly 50 ft	306	88.4 (84.7–91.5)	86	86 (78.1–91.8)	Pearson’s chi-square with Yates’s correction = 0.235 *P* = 0.628
	> 50 ft	40	11.6 (8.5–15.7)	14	14 (8.2–21.9)	
Drinking habit	Fresh water	346	100 (99.1–100)	100	100 (99.1–100)	
Eating habit	Rarely eat outside the house (cafeteria)	78	22.5 (18.4–27.2)	33	33 (24.3–42.6)	Pearson’s chi-square with Yates’s correction = 3.995 *P* = 0.046
	Sometimes eat in cafeteria	268	77.5 (72.8–81.6)	67	67 (57.4–75.7)	
Hand washing before eat	sometimes wash with soap and water before eating	199	57.5 (52.3–62.7)	58	58 (48.2–67.4)	Pearson’s chi-square with Yates’s correction = 0.000 *P* = 1.000
	Rarely/Not washing hand with soap and water before eating	147	42.5 (37.3–47.7)	42	42 (32.6–51.8)	
Hand washing after defecation	sometimes wash with soap and water	340	98.3 (96.4–99.3)	98	98 (93.5–99.7)	Pearson’s chi-square with Yates’s correction = 0.000 *P* = 1.000
	rarely/Not washing with soap water	6	1.7 (0.7–3.6)	2	2 (0.3–6.5)	
Nails cutting habit	Regularly cutting nails	1	0.3 (0.000–1.4)	0	0	T-test (trend test based on contrasts) [Barlow et al.]: chi-sq. = 0.267 [DF = 1] 2-sided *P* = 0.605 for trend in a given direction: *P* = 0.303
	sometimes cutting nails	238	68.8 (63.8–73.5)	68	68 (58.4–76.6)	
	cutting nails after a long time	107	30.9 (26.2–35.9)	32	34 (23.4–41.6)	

The comparison of hygienic practices among the patients and the control group showed no statistical differences as shown in the Table 2.

### Knowledge, attitude and practice towards ALA among the patients

None of the patients nor the controls had the knowledge of the name of the disease condition, aetiological agent, transmission route, predisposing factors and prevention and control measures. Both the patients and control group (70% and 80% respectively) believed that eating well was the way to prevent any liver disease caused by alcohol consumption. Similarly, both groups did not adopt any particular preventive measures in order to prevent the condition, ALA.

### Seasonal variation of the disease

**Fig. 2:**
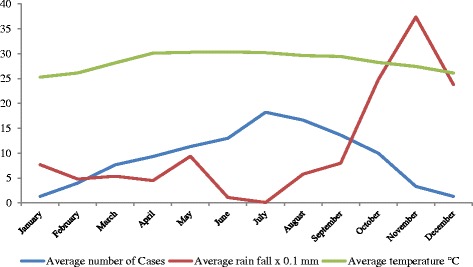
Comparison of number of cases admitted to the Teaching Hospital, Jaffna, with average monthly climatic data during the study period

The monthly incidence of ALA showed the same pattern as the monthly average temperature (Fig. 2) during the study period. The number of cases was high during the dry season compared to the wet or rainy season (Fig. 2). Although the number of cases declined from 2012 to 2015, each year showed the same pattern of seasonality. At the same time, the relative humidity did not show any pattern with the disease.

### Seasonal variation of alcohol consumption

**Fig. 3:**
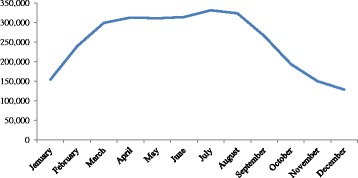
Monthly mean value of total toddy sold in the district

As shown in Fig. 3, alcohol consumption showed a marked seasonality; specifically median consumption was high in the middle months of the year i.e. in the hot season.

### History of alcohol consumption

All patients (100%) had a positive history of consumption of alcohol. The majority (79.2%) were in the habit of consuming toddy and arrack, depending on the money availability. Those who were consuming toddy, consumed palmyrah toddy (100%). The majority (*n* = 245, 70.8%) stated that they consumed alcohol daily (Table 3). As shown in Table 4, among the patients who consumed alcohol daily, 63.7% and 31.4% were of the age groups 41–50 years and 31–40 years, respectively.

**Table 3:** Detail description of alcohol consumption habit among ALA patient compared with individual who are *E. histolytica* antibody negative individuals

Factors	Category	Patients (Cases)		*E. histolytica* serum antibody negative individuals (controls)	
		*N* = 346	% with CI	*N* = 100	% with CI
Alcohol drinking habit	yes	346	100 (99.1–100)	100	100 (99.1–100)
Type of alcohol	Only Toddy	72	20.8 (16.8–25.3)	94	94 (87.9–97.5)
	Toddy + arrack	274	79.2 (74.7–83.2)	6	
Type of toddy	Palmyrah	346	100 (99.1–100)	100	100 (99.1–100)
Frequency of drinking	Daily	245	70.8 (65.9–75.4)	2	6 (2.5–12.1)
	4–6 times a week	101	29.2 (24.6–34.1)	20	20 (13.0–28.7)
	2–3 times a week	0	0	10	10 (5.2–17.1)
	2–4 times a month	0	0	68	68 (58.4–76.6)

**Table 4:** Comparison of significantly differing factors between ALA patients and *E. histolytica* antibody negative individuals

Factors	Category	*E. histolytica* serum Antibody (IgG)		Statistics
		Positive (cases) *N* = 100	Negative (Control) *N* = 100	
Age group	21-30 yrs	0	18	Chi- Square-56.63; Df-3; *P* < 0.001
	31–40 yrs	29	29	
	41-50 yrs	66	23	
	51-60 yrs	5	30	
Type of alcohol	Only toddy	27	94	Chi- square- 99.92; Df-1; P < 0.001,OR-4.5, 95%CI: 1.94–11.96
	Toddy + Arrack	73	6	
Frequency of drinking	Daily	70	2	Chi Squre-1.481Ea Df-3 *P* < 0.0001
	4–6 times a week	30	20	
	2–3 times a week	0	10	
	2–4 times a month	0	68	

### Comparison of some risk factors among patients and control group

Statistical analysis revealed that age, type of alcohol and frequency of consumption of alcohol was the associated factors for ALA (Table 4). In further analyses (adjusted for age and group of daily consumers and who consumed 4–6 times in a week amalgamated as one group and compared with amalgamated group of 2–3 times a week and 2–4 times in month) revealed that the frequency of alcohol consumption (daily consumers and those who consumed 4–6 times a week) (AOR: 701.3, 95%CI: 103.5 - infinitive) and type of alcohol (toddy and arrack) (OR-4.5, 95%CI: 1.94–11.96) were the potential risk factors.

## Discussion

Since 1985, ALA has been a public health burden second only to malaria in terms of parasitic infections in Jaffna district (personal communication with the clinicians), northern Sri Lanka. We found that the annual admission rate of ALA in 2012, 2013, 2014, 2015 were 9.0, 7.0, 4.0, and 3.0 per 10,000 total admissions at TH, Jaffna, respectively. Even though a decreasing trend was seen, ALA is still responsible for significant morbidity.

In 1985, the reported rate of ALA at TH, Jaffna was 5.9% of total hospital admissions [2]. Taken together with the present findings, this indicates that Jaffna has remained persistently endemic. Similarly, Rajasuriya and Nagaratnam [7] reported in 1962 that 1.5% of total hospital admissions in the General Hospital Colombo were due to ALA, and Ramachandran et al. [8] reported that 3.5% of total hospital admissions in Negombo Hospital were due to ALA, which were little lower than that of Jaffna. Although there are no current data available from other parts to compare with our findings, clinicians report that ALA is now rarely seen in other parts of Sri Lanka. Hence, our present findings are more or less in agreement with the study carried out in the same locality [2] 25 years earlier.

Marked male predominance (98.4%) was observed in our study as well as in other surveys carried out in Sri Lanka and elsewhere [3–5, 9, 10, 14–28]. This may be due to the differences in alcohol consumption between the two sexes, as well as other contributory factors. Similar patterns have been observed in most countries where amoebiasis is endemic. Where the disease was reported among travellers, and the country is non-endemic, male preponderance was little bit less i.e. 54% in Spain [27] and 78% in France [28].

Similarly, the age distribution (31–50) also tallies with reports around the world [3–5, 7, 14, 17–21, 26–28, 30]. Further, frequency of the consumption of alcohol and type of alcohol were identified as the potential risk factors (Table 4). Due to the limitation of small sample size, we were unable to explore the risk factors further. A further, well designed prospective (cohort) study is recommended to explore this finding.

It has been suggested, based on animal models (hamster), that the male hormone testosterone could be a host factor that favours the development of ALA [28]. The positive role of testosterone is one of the explanations that middle-aged men were mostly affected; with increasing age (after 50 years) the incidence declines with the reduction of testosterone levels [28].

Further, it is a well-known fact that iron is a crucial component of *E. histolytica* enzymes [29]. In vitro experimental studies have showed that iron is a vital growth factor for *E. histolytica* [30]. Regular menstrual blood loss in females of reproductive age, may lead to lower iron stores [30] which are mainly in the liver [31]. Further, Ralph Warren and John Kempston [32] have also proposed that alcoholic hepato-cellular damage in males and the protective effect is mainly due to the iron deficient anaemia or protective hormonal factors in women of child bearing age. However, alcohol consumption may be lower among females due to cultural reasons. Interestingly, in our study, the few females who had ALA also had a history of consuming toddy.

Makkar et al. [30] have suggested that high content of iron and a diet rich in carbohydrate in habitual consumers of alcohol may predispose to invasive amoebiasis, leading to ALA. Further, age predilection and gender bias in the formation of ALA is also based on the high alcohol intake [18].

Apart from age and sex, the other associated risk factor identified in our study was consumption of alcohol (100%). A majority of the researchers working on ALA have reported the habit of alcohol consumption, particularly in endemic areas [3–5, 10, 14–21].

The percentage of patients who consumed alcohol varied from 50% in Bangladesh [10] to 100% in Jaffna, Sri Lanka [5]. Our previous findings (100%) in this locality still stand in this regard [5].

Ghosh et al. [18] have reported that alcohol suppresses the function of Kupffer cells in the liver which has the important role in clearing the amoeba.

Further, Raja and Karthick [16] suggested that the invasive capacity of *E. histolytica* is facilitated by alcohol which is harmful to the liver and by nutritional deficiencies in alcoholics, thus leading to a higher incidence of ALA in alcoholics. Further, they reported that duration and quality of alcohol consumed also play an important role in the higher incidence of liver abscess and therefore alcoholism is the most important predisposing factor [16].

Meanwhile, Mukhopadhyay et al. [17] suggested that alcohol can predispose to ALA in the following manner, a) hepatic damage by alcohol, b) habitual consumers often neglect meals resulting in lowered body resistance and suppression of liver function, c) liquor prepared locally with no regard for asepsis has a large population of amoeba in it and d) immunity in chronic alcoholics is depressed. In an earlier report, Hai et al. [9] reported the involvement of toddy in predisposing to ALA, but noted that the mechanism was unclear. On the contrary, Siddiqui et al. [10] argued that indigenous alcohol has much more association with the development of pyogenic liver abscess. Meanwhile, Ramachandran [33] and Fernando et al. [3] strongly believed toddy was the medium for the transmission of *E. histolytica*.

At the same time, Raja and Karthick [16] further reported that though alcoholism seems to be the predisposing factor, it has no role in the aetiology.

Further, Makkar et al. [30] also disagree with the statement that “toddy becomes susceptible because of the large dose of *E. histolytica* ingested with the drink”. They argued that ALA seems to be common in alcoholics irrespective of the type of alcohol used.

In our study too, we were unable to demonstrate the infective stage of *E. histolytica* from toddy. Further, we found that 79% of the patients consumed toddy as well as arrack depending on the money availability which was found as an independent risk factor.

We found that the peak of the disease was correlated with the dry seasons (Fig. 2). The number of cases increased from March up to July and started to decline until September. A similar finding was observed in a study carried out last year in India [11], where they have reported that the transmission predominantly occurred late summer to early rainy seasons.

There is a belief among the consumers that the toddy has a cooling effect on the body during the hot dry season. Therefore, this may be the reason for increased intake of toddy during the dry season and for the increasing rate of the disease which was further supported by the increased sales of toddy during the dry season.

## Conclusion

Though the number of cases has declined in recent years, amoebic liver abscess still remains an important public health problem in Jaffna district, northern Sri Lanka. Middle aged males who reported heavy consumption of alcohol, and lived mainly in rural areas, were mostly affected by this condition. The incidence was highest in the dry season. Statistical analysis showed the type of alcohol and the frequency of consumption to be the potential risk factors. However, none of the toddy samples demonstrated *Entamoeba histolytica* cysts. Hence, establishing the route of transmission of the parasite is of great importance. Moreover, all patients and controls were found lacking in knowledge, attitude and practice towards ALA. Therefore, conducting awareness programs through mass media and community level to the public who are at risk is essential, in order to eliminate the disease.

## Additional file

Additional file 1: English language version of the questionnaire. The English language version of the questionnaire used to collect the following information a) general socio demographic data b) personal hygienic practices and alcohol consumption history and c) the knowledge, attitude and practices in relation to ALA. (DOCX 18 kb)

## Abbreviations

ALA: Amoebic liver abscess
AOR: Adjusted odds ratio
CI: Confidence interval
Df: Degrees of freedom
G.C.E O/L: General certificate of education ordinary level
KAP: Knowledge, attitude and practice
LKR: Sri Lankan rupees
OR: Odds ratio
SPSS: Statistical package for the social sciences
TH: Teaching hospital
