# Supplementary material for: Epidemiology and factors associated with amoebic liver abscess in northern Sri Lanka
Source: BMC Public Health. 2018 Jan 10;18:118. doi: 10.1186/s12889-018-5036-2 (PMC5761098; doi:10.1186/s12889-018-5036-2)
Supplement: Additional file 1: — English language version of the questionnaire. The English language version of the questionnaire used to collect the following information a) general socio demographic data b) personal hygienic practices and alcohol consumption history and c) the knowledge, attitude and practices in relation to ALA. (DOCX 18 kb) [file 12889_2018_5036_MOESM1_ESM.docx]

Additional file 1: English language version of the questionnaire

**A study on seroepidemiology and molecular characterization of *Entamoeba histolytica***

**among amoebic liver abscess patients, attending to the Teaching Hospital, Jaffna**

Mark “√” for the appropriate response

Serial Number…………………….

1. Name of the patient…………………………………………………………………………
2. Address of the patient …………………………………………………………………………

………………………………………………………………………………………

1. Sex Male……………………………… Female: ………………

4. Age

| < 17 yrs |  |
| --- | --- |
| 18- 20 yrs |  |
| 21- 30 yrs |  |
| 31- 40 yrs |  |
| 41- 50 yrs |  |
| 51- 60 yrs |  |
| >60 yrs |  |

5. Educational Level

| No school education |  |
| --- | --- |
| Up to grade 5 |  |
| Grade 6 to G.C.E. O/L |  |
| Studied G.C.E. A/L |  |
| Higher Education |  |
| Others (specify) |  |

6. Occupation

| No Job |  |
| --- | --- |
| Laborer |  |
| Farmer |  |
| Fisherman |  |
| Self employed |  |
| Teacher/clerk/management assistant |  |
| Executive service |  |
| Other (specify) |  |

7. Ward Number…………………

8. BHT Number……………………

9. Latrine facilities

| None |  |
| --- | --- |
| Pit |  |
| Water seal |  |
| Others (specify) |  |

10. Source of drinking water

| Protected well |  |
| --- | --- |
| Unprotected well |  |
| Tube well |  |
| Public/street tap |  |
| Other (specify |  |

11. Do You drink any kind of alcohol

| No |  |
| --- | --- |
| Ex drinker ( no alcohol last 6 months) |  |
| Yes |  |

If Yes

12. What type of alcohol you usually drink (or did drink)

| No preference |  |
| --- | --- |
| Only arrack |  |
| Only Toddy |  |
| Only Beer |  |
| Only “kasippu” |  |
| Toddy + arrack |  |
| Toddy + “kasippu” |  |
| Toddy + arrack + “kasippu” |  |
| Other (specify) |  |

13. If you consume toddy

| Coconut |  |
| --- | --- |
| Palmyra |  |

14. Frequency of drinking habit

| 2- 4 times a month |  |
| --- | --- |
| 2- 3 times a week |  |
| 4- 6 times a week |  |
| Daily |  |
| Others (Specify) |  |

15. Do you aware what kind of condition you have Yes …………….No……………

16. Have you taken any medication for this condition before coming here Yes…………No

17. Have you had this kind of condition before Yes…………No………….

18. If yes When……………………………………………………………….

19. Do you know what causes this condition………………………………………………….

20. do you know how this condition is spread……………………………………………….

21. Did you have the history of diarrhea (within 6 month) Yes……………No………….

22. If yes, when ……………………

23. Type of diarrhea

Watery………………Blood & Mucous…………Other (specify)………………
